# Supplementary material for: Hepatocyte mitochondria-derived danger signals directly activate hepatic stellate cells and drive progression of liver fibrosis
Source: Nat Commun. 2020 May 12;11:2362. doi: 10.1038/s41467-020-16092-0 (PMC7217909; doi:10.1038/s41467-020-16092-0)
Supplement: Supplementary file 1 — Supplementary Information [file 41467_2020_16092_MOESM1_ESM.pdf]

Supplementary Information file

Hepatocyte mitochondria-derived danger signals directly activate hepatic stellate cells and drive progression of liver fibrosis by Ping An et al.

Corresponding author: Yury Popov

Email: ypopov@bidmc.harvard.edu

This PDF file includes:

Supplementary Figures 1 to 9

Supplementary Tables 1 to 6

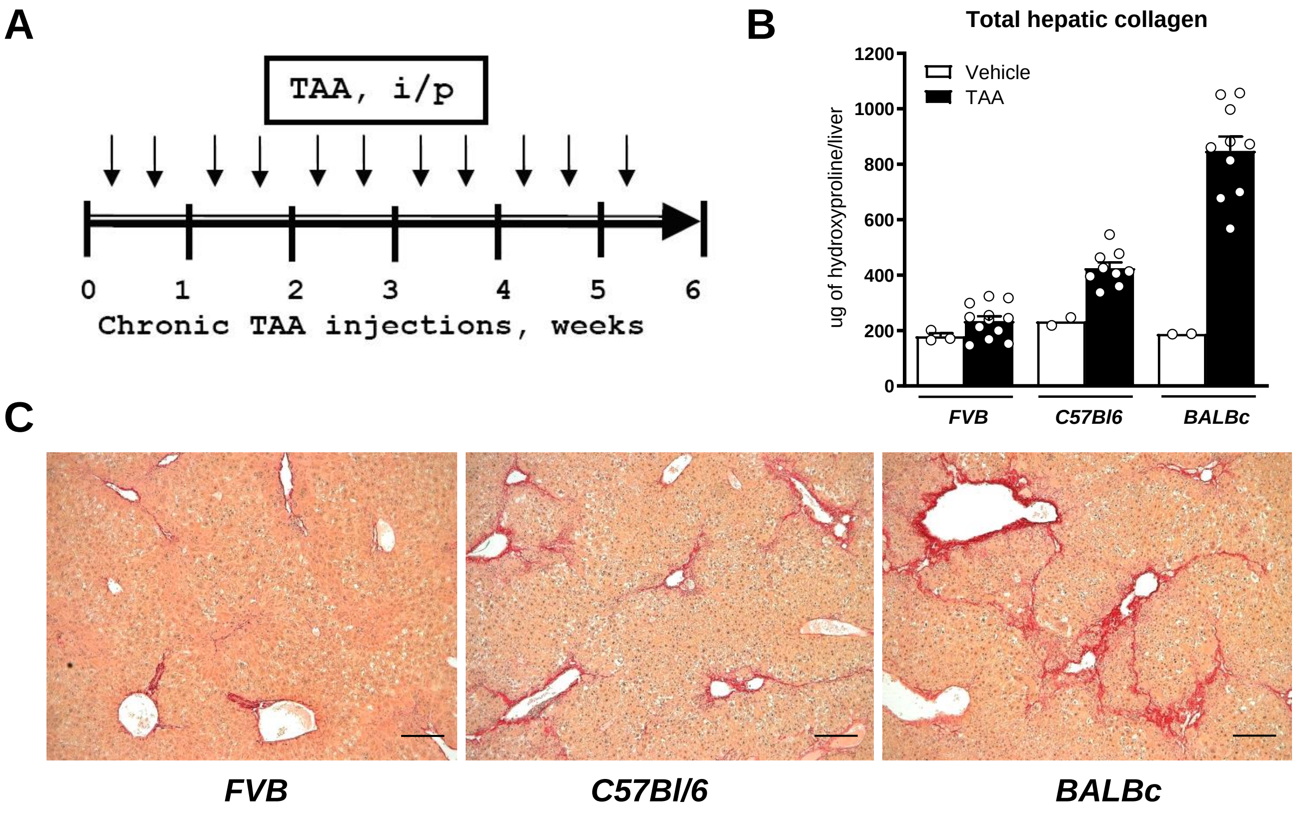

Supplementary Figure 1. Inbred mouse strains demonstrate significant differences (BALBc>C57Bl6>FVB) in susceptibility to liver fibrosis in a model of chronic liver injury induced by repeated thioacetamide injections. FVB demonstrate resistance to fibrosis, BALB/c strain develop most significant fibrosis and C57Bl/6 mice showed intermediate susceptibility, generally reproducing previous data on inbred strain susceptibility to liver fibrosis in response to another hepatotoxin, carbon tetrachloride (see reference 2 by Hillenbrand et al, 2002 in the main manuscript). **A.** Experimental design of chronic TAA-induced liver fibrosis model in inbred mice: 2 injections per week, with sacrifice in lieu of final injection at 6w of induction. **B.** Collagen deposition in livers of FVB, C57Bl/6 and BALBc mice receiving chronic TAA injections. Hepatic collagen content was determined biochemically via hydroxyproline (HYP) determination as described in M&M. Total HYP (µg/whole liver) was calculated from individual liver weights and respective relative HYP values. **C.** Connective tissue staining (bar, 100µm). Ctrl: non-fibrotic control group (n=3/2/2) that received vehicle (saline) only; TAA: fibrotic mice treated with TAA for 6 weeks (n=12/9/10 for FVB/C57/BALBc, respectively). Data are expressed as means ±SEM. Source data are provided as a Source Data file.

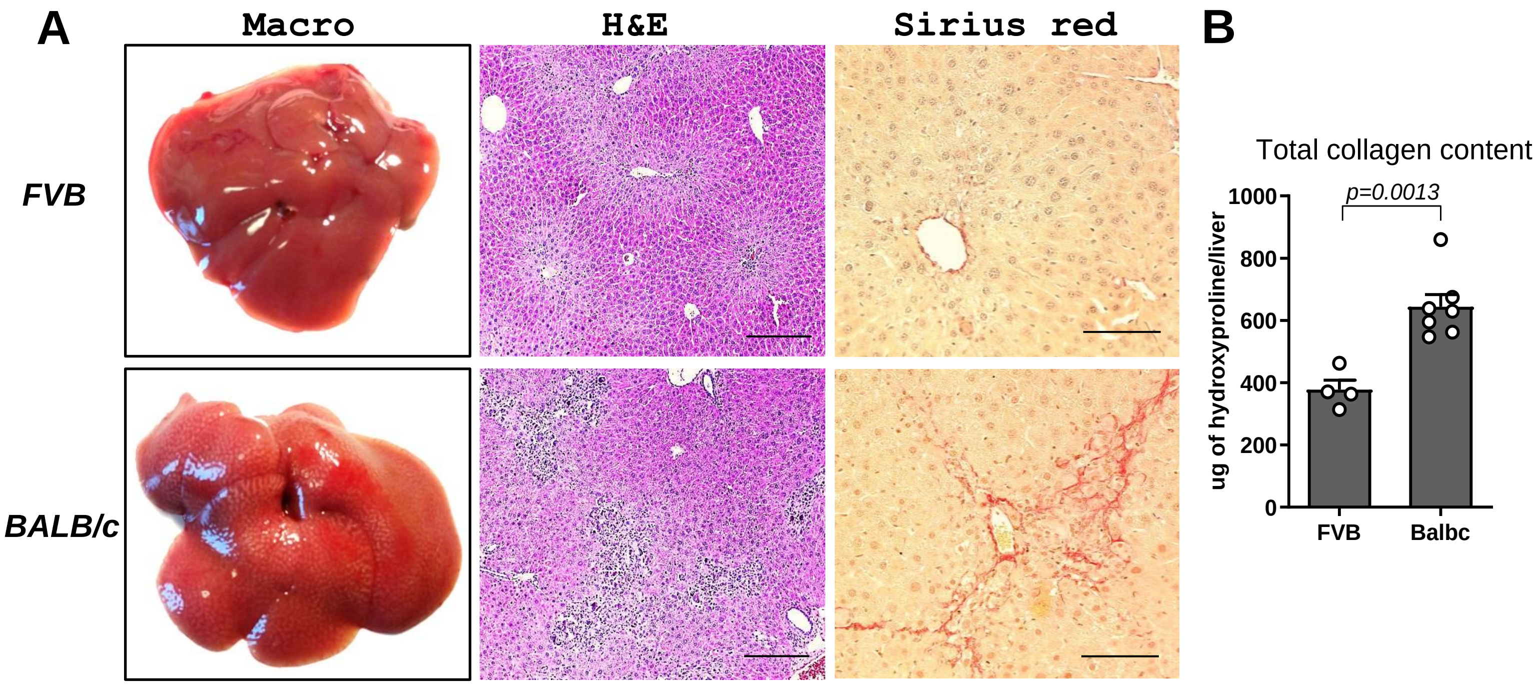

Supplementary Figure 2. Inbred strains demonstrate dramatic differences in fibrotic responses to a single sub-lethal liver injury induced by CCl4. A. Macroscopic appearance of representative livers from FVB and BALB/c mice 8 days post-CCl4 injury (left panel). After a single dose CCl4 administration, FVB livers completely recovered by day 8 and appear virtually indistinguishable from healthy uninjured liver, while BALB/c livers demonstrate increased turgor, stiff consistence and strong whitish pattern indicative of incomplete repair. Histological examination suggests necrotic masses are cleared in fibrosis-resistant FVB mice but persist in fibrosis-susceptible BALB/c mice (representative low-magnification images at day 8, hematoxylin/eosin, middle panel; bar, 100µm). Connective tissue staining demonstrates significant pericentral deposition of collagen in BALB/c mice (right panel; bar, 50µm). No collagen deposition occurs in FVB mice without macrophage depletion. **B.** Collagen deposition in livers of FVB and BALB/c mice at day 8 after CCl4-induced liver insult (FVB, n=4; BALB/c, n=7). Data are expressed as means ±SEM. *P* value as indicated (two-tailed, unpaired t-test). Source data are provided as a Source Data file.

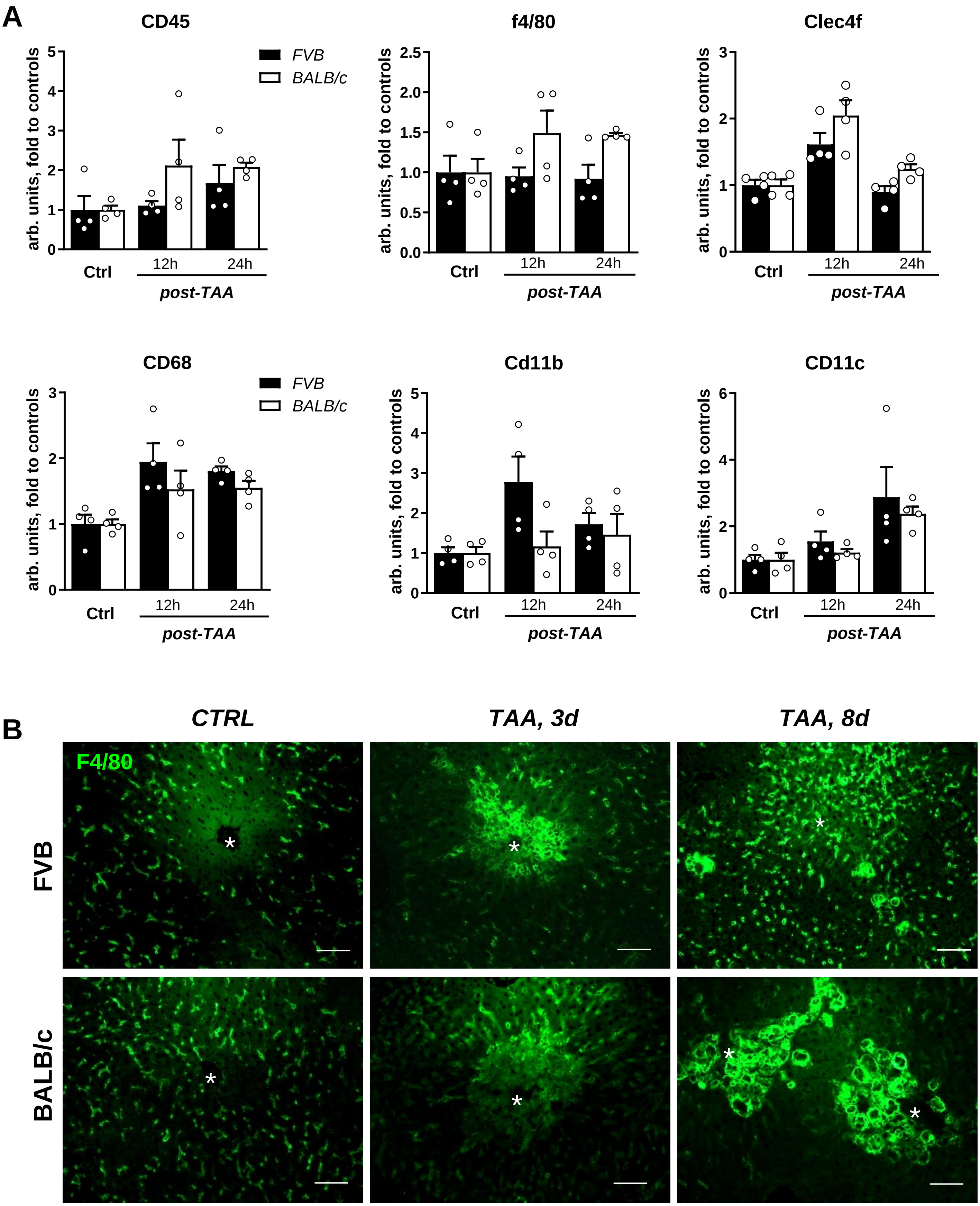

Figure continued on next page

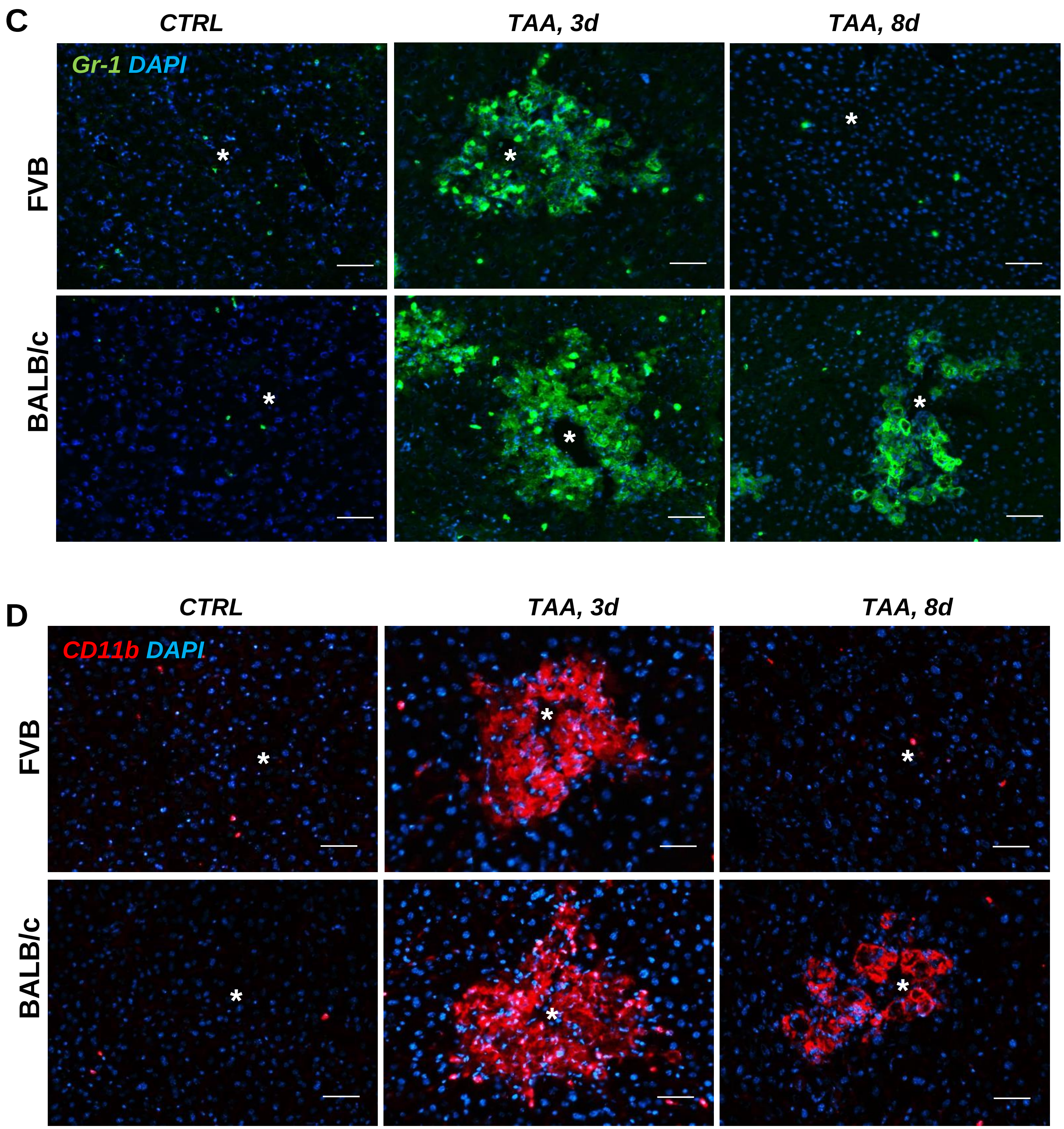

Supplementary Figure 3. Temporospatial strain differences in myeloid/monocytic infiltration in fibrosis-resistant FVB strain versus fibrosis-susceptible strain BALB/c. (A) No significant strain differences in immune cell subsets recruitment in early stages of liver injury in FVB and BALB/c mice (12-24h). Quantitative TaqMan RT-PCR in total hepatic RNA extracts (n=4) for immune cell markers CD45 (pan-leukocyte), F4/80 (pan-macrophage), Clec4F (resident macrophages/Kupffer cells), CD68&CD11b (infiltrating

macrophages/monocytes), and CD11c (dendritic cells). Data are expressed as means±SEM, fold to respective strain controls relative to HPRT-1 as a housekeeping gene (n=4 individual mice per bar, $p>0.05$ between strains compared at each time-points, ANOVA followed by Tukey post-test). Data are expressed as means ±SEM. (B) Immunofluorescent staining for pan-macrophage marker F4/80 reveals significant strain differences in macrophage infiltration at intermediate (3 days) and late (8 days) stages post-TAA injury (representative images at original magnification, 200x. * indicates central vein). Note delayed migration of F4/80+ Kupffer cells into pericentral necrotic areas at day 3, and persistence of massive macrophage infiltrates at day 8 in fibrosis-susceptible BALB/c strain after single injury. Immunofluorescent staining for myeloid markers Gr-1 (C) and CD11b (D) reveals significant differences in myeloid cell infiltration at late (8 days) stages of repair post-TAA injury. While there are few Gr-1+ or CD11b+ cells found in healthy liver, massive infiltration of Gr-1+ and CD11b+ cells into pericentral necrotic areas is observed at day 3 after injury in both FVB and BALBc strains. Gr-1/CD11b+ infiltrates persists even at day 8 in fibrosis-susceptible BALB/c strain. In contrast, the fibrosis-resistant FVB livers demonstrate complete resolution of immune infiltrates by day 8. Representative images shown at original magnification, 200x. * indicates central vein. Bar, 50μm. Source data are provided as a Source Data file.

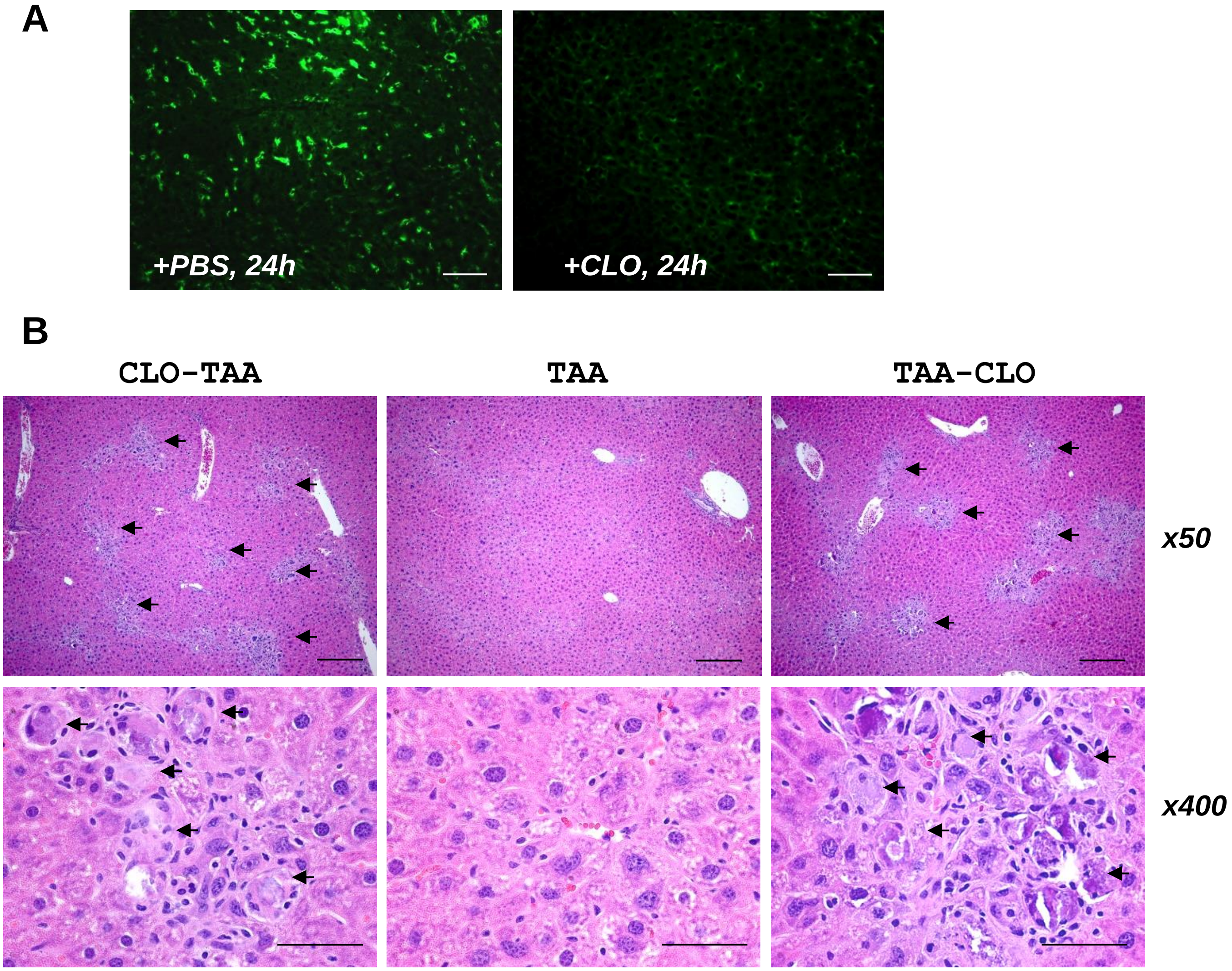

Supplementary Figure 4. Macrophages depletion using clodronate-loaded liposomes efficiently eliminates resident liver macrophages and impairs clearance of dead hepatocytes after hepatotoxic insult. A. Single i/p injection of clodronate-loaded liposomes (CLO, 10ul/g) efficiently depletes resident hepatic macrophages. 24h after CLO injection, virtually all liver macrophages are eliminated. Immunofluorescent detection of macrophage marker F4/80, original magnification, 200x (bar, 50μm). B. Macrophage depletion either before or after TAA-induced hepatotoxic injury in FVB mice prevents efficient clearance of dead hepatocytes, with necrotic masses (arrows) persisting 8 days after TAA injection. Necrotic areas are completely cleared 8 days after receiving TAA (TAA, middle column) in FVB mice not treated with CLO. Resident macrophages were depleted by single injection of clodronate liposomes 24h prior to TAA administration (CLO-TAA, left column). Infiltrating and resident macrophage depletion was performed when clodronate liposomes were injected 24 hours after TAA injection (TAA-CLO, right column). Bar, 100μm (middle row) and 20μm (lower row). All mice were sacrificed and evaluated at day 8 post-TAA, images are representative of 4 independent animals.

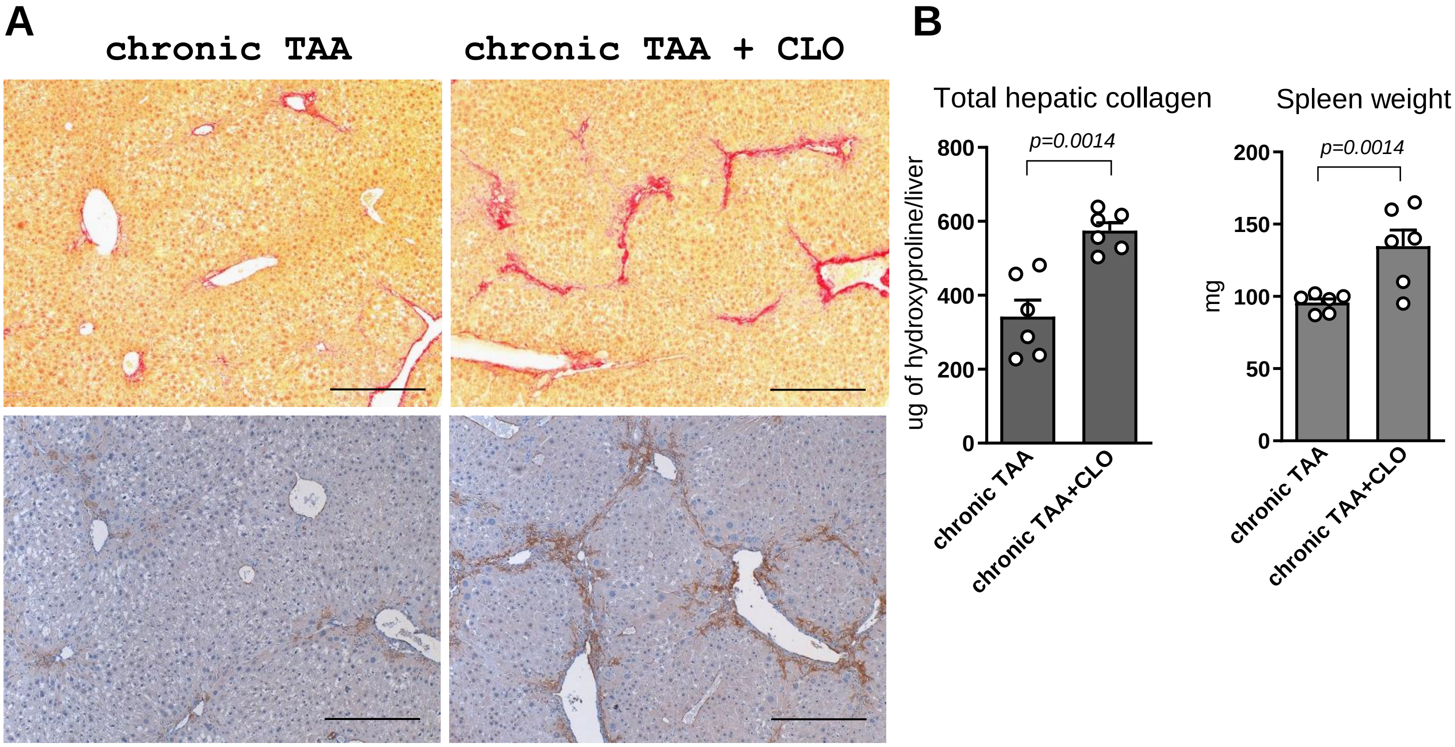

Supplementary Figure 5. Selective depletion of phagocytic macrophages during chronic TAA-induced liver injury leads to significant hepatic fibrosis development in fibrosis-resistant FVB strain. Chronic liver injury was induced using chronic i/p injection of TAA for 6 weeks in FVB mice. Selective depletion of macrophages was achieved by weekly injections of clodronate-loaded liposomes (chronic TAA+CLO) concurrently with TAA administration in FVB mice. Control group (chronic TAA) received PBS-loaded liposomes instead of CLO. **A.** Connective tissue staining (upper panel) demonstrates development of bridging fibrosis in fibrosis-resistant strain (FVB mice) with macrophage depletion (chronic TAA+CLO). α-SMA staining (lower panel) indicates significant accumulation of activated hepatic stellate cells within fibrotic septa. No significant fibrosis and only minimal α-SMA signal observed in FVB mice receiving control liposomes (chronic TAA). Bar, 50μm. **B.** Significant hepatic collagen deposition and splenomegaly in mice receiving clodronate-loaded liposomes as quantified via hepatic collagen content. Data are expressed as means ±SEM (n=6 individual mice per group). *P* value as indicated (two-tailed, unpaired t-test). Source data are provided as a Source Data file.

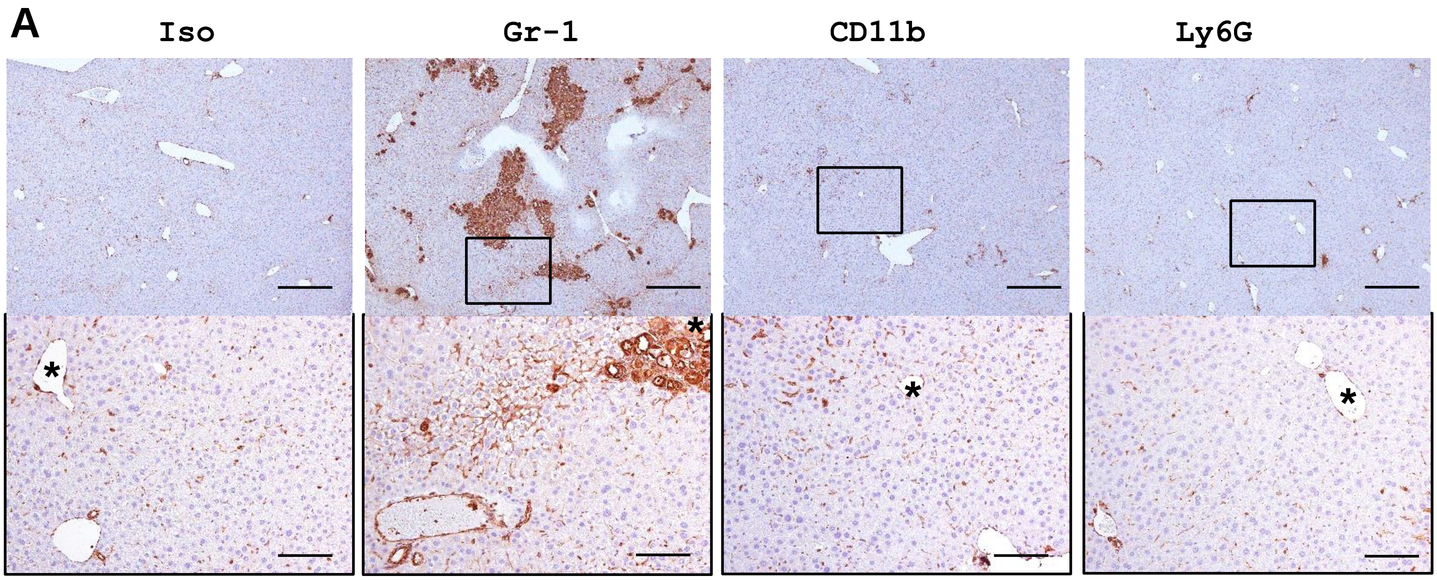

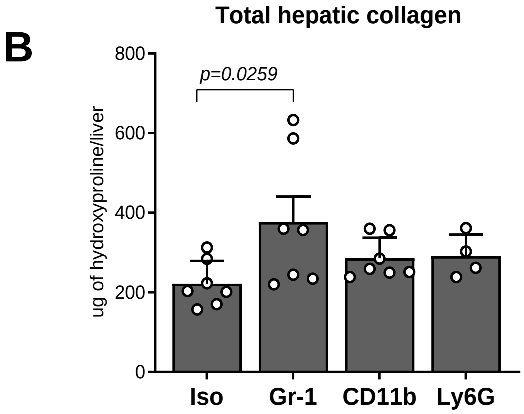

Supplementary Figure 6. Impact of post-injury myeloid cell subsets depletion on hepatic stellate cell activation marker α-SMA expression and hepatic collagen deposition. Fibrosis-resistant FVB mice were administered cell subset specific antibody to deplete myeloid cells (Gr-1 mAB RB6-8C5, 200ug/mouse), monocytes (CD11b mAB M1/70, 200ug/mouse), isotype control (Iso, LTF-2 IgG2b, 200ug/mouse) or granulocytes/neutrophils (Ly6G mAB 1A8, 500ug/mouse) on 1st, 3rd and 5th day after TAA injections (n=7/7/7/4 animals for Iso/Gr-1/Cd11b/Ly6G groups, respectively). (**A**) Immunohistochemistry for α-SMA was performed on day 8 (upper row, representative low-magnification images shown, x50, bar 100μm and lower row, blow-up images at x200, bar 200μm). While isotype-control livers demonstrate minimal α-SMA immunopositivity, Gr-1+ myeloid cell depletion leads to massive activation of stellate cells in fibrotic septa-like pattern. Note that strong signal in necrotic area is a staining artifact and should not be considered. Antibody depletion of CD11b+ or Ly6G+ cell subsets did not lead to stellate cell activation. (**B**) Hepatic collagen deposition as determined biochemically via hydroxyproline content. Data are mean±SEM. * $p<0.05$ compared to isotype-treated controls (ANOVA with Dunnett's post-test). See also main Fig. 4E for additional data on impaired hepatocyte clearance and histological fibrosis in this experiment. * indicates central vein. Source data are provided as a Source Data file.

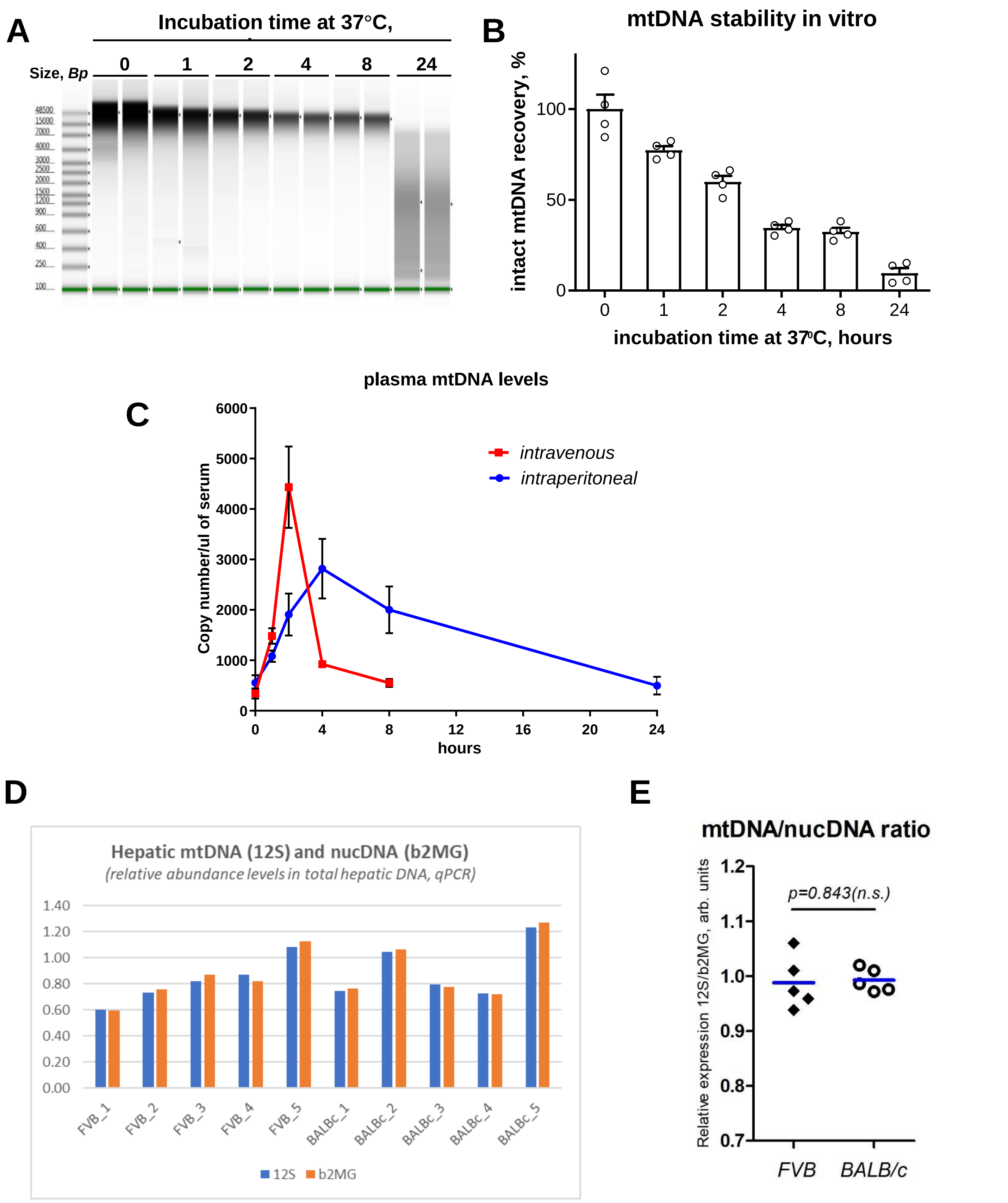

Supplementary Figure 7. Analysis of mito-DAMPs stability in vitro and systemic exposure to exogenously administered mito-DAMPs in vivo. Freshly prepared mito-DAMPs preparations were incubated at 37C with a

low-speed agitation and sampled at 0, 1, 2, 4, 8, 24 hours and DNA isolated to assess mtDNA integrity (two individual preparations shown). (**A**) DNA microgel image (B) and densitometry analysis showing progressive decline in recovered intact DNA over time (**B**, expressed as percent of initial concentration, n=4). (**C**) mito-DAMPs (9.5 ug of mtDNA/mouse) isolated from liver mitochondria were injected into tail vein or peritoneal cavity of healthy FVB mice, and mtDNA levels in plasma measured 1, 2, 4 8 and 24 hours later (n=2-5 per time point). (**D)** Relative abundancy of mitochondrial DNA and nuclear DNA template copies in total liver DNA of healthy untreated FVB and BALB/c mice, as determined by TaqMan qPCR for 12S and b2MG genes, respectively (n=5, each bar represent the individual value for each mouse). (**E**) mtDNA/nucDNA ratio demonstrate no difference in hepatic mtDNA genome copy numbers between studied inbred mouse strains. Blue line indicate group mean, two-tailed p value was determined using unpaired t-test (n.s., not significant, n=5). Relative abundance of DNA template was calculated using internal calibration and second derivative maximum method. Data are expressed as means ±SEM. Source data are provided as a Source Data file.

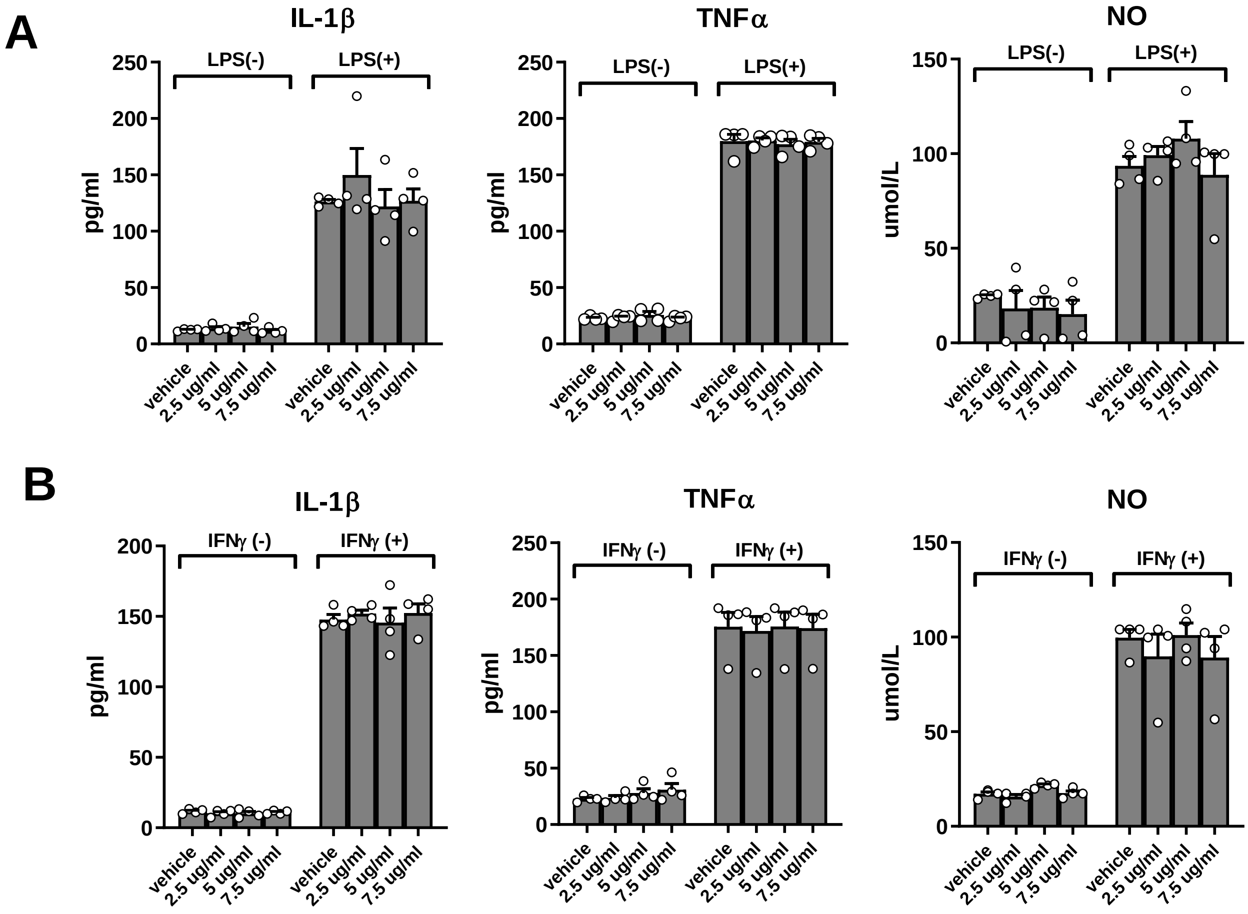

Supplementary Figure 8. Primary resident liver macrophages (Kupffer cells) are not activated by mito-DAMPs in vitro. Freshly isolated murine Kupffer cells in 12-well plates (1×10^6/well) were incubated for 24h in vitro with increasing concentration of mito-DAMPs (corresponding to 2.5-7.5 ug of mtDNA/ml) prepared from purified liver mitochondria, with or without LPS or INFγ stimulation. **(A)** IL-1b, TNFa and NO secretion into cell culture supernatant upon exposure to mito-DAMPs at baseline and 100 µg/ml LPS co-stimulation **(B)** IL-1b, TNFa and NO secretion into cell culture supernatant upon exposure to mito-DAMPs at baseline and upon 100U/ml IFNy co-stimulation. Results are representative of two individual cell isolations with 4 biological replicates/condition each. Data are expressed as means ±SEM. Source data are provided as a Source Data file.

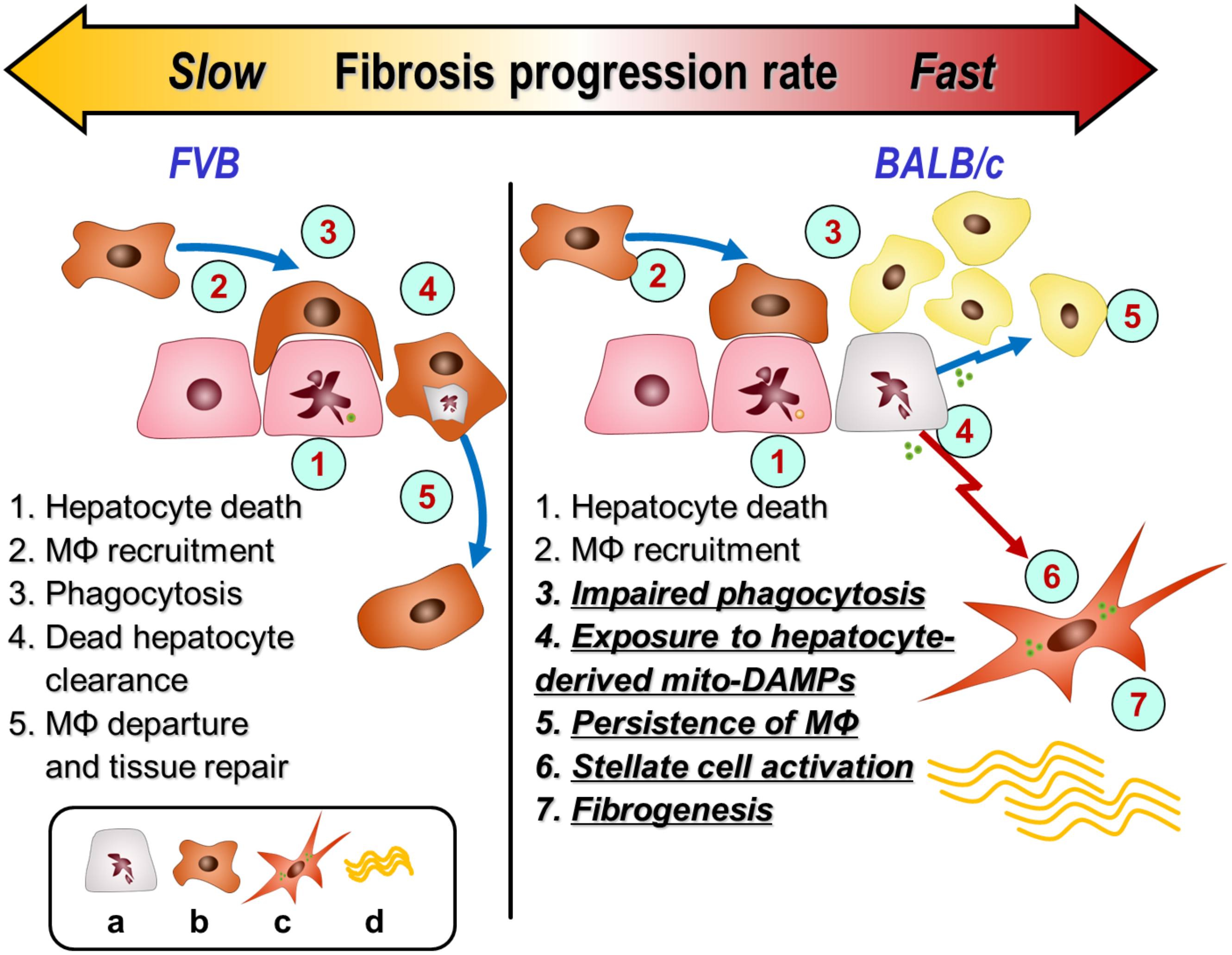

Supplementary Figure 9. Proposed pathophysiological basis of susceptibility to post-necrotic liver fibrosis and cirrhosis. In fibrosis-resistant mouse strain FVB (left), hepatocyte death (1) elicits resident macrophage and Gr-1+ myeloid cell recruitment and (2) phagocytosis of dead hepatocytes and (3) their rapid clearance, followed by (4) macrophage departure and scarless hepatic tissue repair. In "rapid fibroser" mouse strain BALBc (right), or when phagocyte function is compromised (e.g. by CLO in resistant FVB mice), macrophage-mediated phagocytosis of dead hepatocyte is impaired (3), which leads to exposure to dead hepatocyte intracellular content (e.g. mito-DAMPs) (4), persistence of infiltrating macrophages (5), and activation of stellate cells that deposit collagen (6,7). (a) necrotic/apoptotic hepatocyte, (b) macrophage, (c) activated hepatic stellate cell, (d) fibrotic matrix.

Supplementary Table 1. Sequences of probes and primers used in real-time PCR and RT-PCR.

Gene	Sequences (5'-3')
**Genomic (mtDNA) 12S (mouse)**	Forward: ACT GGA AAG TGT GCT TGG AA<br>Reverse: GTG TAG GGC TAG GGC TAG GA<br>Probe: AAG CAT CTG GCC TAC ACC CAG AAG A
**Genomic (mtDNA) 16S (mouse)**	Forward: TTG TAC CTT TTG CAT AAT GAA CTA ACT<br>Reverse: TTT GCC ACA TAG ACG AGT TGA<br>Probe: AAA CCC CGA AAC CAA ACG AGC TAC C
**Genomic (nuclear) β2MG (mouse)**	Forward: CAG ACT CTG CGA TGT TTC CA<br>Reverse: CTA CCT CTG CCT CCC AAG TG<br>Probe: AAT AAC CTT AAA GGT CGC CGG GCA G
**Genomic (mtDNA) D-loop (human)**	Forward: CAC AGC CAC TTT CCA CAC AG<br>Reverse: TGG TTA GGC TGG TGT TAG GG<br>Probe: CGC TTC TGG CCA CAG CAC TTA AAC A
**TGFβ1 (mouse)**	Forward: AGA GGT CAC CCG CGT GCT AA<br>Reverse: TCC CGA ATG TCT GAC GTA TTG A<br>Probe: ACC GCA ACA ACG CCA TCT ATG AGA AAA CCA
**Collagen α1(I) (mouse)**	Forward: TCC GGC TCC TGC TCC TCT TA<br>Reverse: GTA TGC AGC TGA CTT CAG GGA TGT<br>Probe: TTC TTG GCC ATG CGT CAG GAG GG
**TIMP-1 (mouse)**	Forward: TCC TCT TGT TGC TAT CAC TGA TTA GCT T<br>Reverse: CGC TGG TAT AAG GTG GTC TCG TT<br>Probe: TTC TGC AAC TCG GAC CTG GTC ATA AGG
**β2MG (mouse)**	Forward: CTG ATA CAT ACG CCT GCA GAG TTA A<br>Reverse: ATG AAT CTT CAG AGC ATC ATG AT<br>Probe: GAC CGT CTA CTG GGA TCG AGA CAT GTG

Supplementary Table 2. Primary antibodies used in immunochemistry and immunofluorescence staining

	Primary antibodies	Application/Dilution
**αSMA(Abcam, ab5694)**	Monoclonal, rabbit anti-mouse	IHC (1:400)
**F4/80 (Serotec, mca497)**	Monoclonal, rat anti-mouse	IF (1:150)
**Desmin (Santa Cruz, sc23879)**	Monoclonal, goat anti-mouse	IF (1:50)
**CD11b (R&D, MAB1124)**	Monoclonal, rat anti-mouse	IF (1:100)
**Gr-1(R&D, MAB1037)**	Monoclonal, rat anti-mouse	IF (1:100)

Supplementary Table 3. Co-localization of fluorescently-labelled apoptotic thymocytes with F4/80 positive and Gr-1 positive cells in the livers of FVB and BALB/c mice (healthy and 48h post TAA-induced injury). 10 randomly chosen HPF were analyzed at x200 magnification per animal. Data are expressed as means ±SEM. #, Two-tailed $p<0.05$ compared to FVB strain at corresponding (healthy or post-injury) condition (unpaired t-test).

**Cell counts/HPF**	**FVB**		**BALB/c**	
	Healthy *(n=3)*	Post-injury *(n=4)*	Healthy *(n=4)*	Post-injury *(n=5)*
**Apoptotic cells count**	19.13±1.44	13.13±1.99	16.20±2.77	6.90±0.39#
**Apoptotic cells/F4/80+**	16.37±1.12	7.83±0.91	14.38±2.50	3.200±0.31#
**Apoptotic cells/F4/80-**	2.77±0.43	5.30±1.13	1.825±0.96	3.700±0.39
F4/80 co-localization (%)	*85.76±1.45*	*61.10±3.16*	*89.62±4.70*	*46.11±4.56#*
**Apoptotic cells/Gr-1+**	1.63±0.3357	0.50±0.07	2.925±0.28#	0.53±0.26
**Apoptotic cells/Gr-1-**	16.80±0.6110	12.40±1.67	14.53±1.08	10.08±0.40
Gr-1 co-localization (%)	*8.42±0.8701*	*4.40±0.21*	*16.40±1.46#*	*4.820±2.13*

Supplementary Table 4. Complete list of quantitative RT^{2}PCR phagocytosis arrays analysis in healthy livers of FVB mice compared to BALBc. qPCR data were normalized to housekeeping gene b2MG (n=4).
†, Two-tailed p value <0.05 (not adjusted, unpaired t-test).

Gene name	Gene Symbol	p-Value	Fold change
Macrophage receptor with collagenous structure	Marco	0.003231†	9.7
GULP, engulfment adaptor PTB domain containing 1	Gulp1	0.009363†	2.38
Integrin alpha M	Itgam	0.029784†	1.94
Ceacam3 Carcinoembryonic antigen-related cell adhesion molecule 3	Ceacam3	0.008293†	1.76
Fc receptor, IgG, low affinity IIb	Fcgr2b	0.118286	1.69
Integrin beta 2	Itgb2	0.01717	1.49
Csf1 Colony stimulating factor 1 (macrophage)	Csf1	0.324158	1.48
C-mer proto-oncogene tyrosine kinase	Mertk	0.062984	1.48
Cd14 CD14 antigen	Cd14	0.242836	1.43
Elmo1 Engulfment and cell motility 1, ced-12 homolog (C. elegans)	Elmo1	0.303455	1.39
Protein S (alpha)	Pros1	0.091469	1.33
Wiskott-Aldrich syndrome homolog (human)	Was	0.293693	1.32
Wingless-related MMTV integration site 5A	Wnt5a	0.491984	1.31
Anxa1 Annexin A1	Anxa1	0.386903	1.3
Dock2 Dedicator of cyto-kinesis 2	Dock2	0.384956	1.29
Yamaguchi sarcoma viral (v-yes-1) oncogene homolog	Lyn	0.074246	1.29
Fc receptor, IgE, high affinity I, gamma polypeptide	Fcer1g	0.353280	1.27
Vesicle-associated membrane protein 7	Vamp7	0.555133	1.27
Signal-regulatory protein beta 1A	Sirpb1a	0.348726	1.23
Clic4 Chloride intracellular channel 4 (mitochondrial)	Clic4	0.745026	1.2
Iqsec1 IQ motif and Sec7 domain 1	Iqsec1	0.912367	1.2
Crk V-crk sarcoma virus CT10 oncogene homolog (avian)	Crk	0.481981	1.19
Integrin alpha V	Itgav	0.447869	1.18
Axl AXL receptor tyrosine kinase	Axl	0.622559	1.17
Crp C-reactive protein, pentraxin-related	Crp	0.379565	1.17
Toll-like receptor adaptor molecule 1	Ticam1	0.984377	1.17
Syntaxin 18	Stx18	0.264770	1.16
Rap guanine nucleotide exchange factor (GEF) 3	Rapgef3	0.958451	1.14
Surfactant associated protein D	Sftpd	0.477474	1.14
Interferon gamma	Ifng	0.496980	1.13
Nucleotide-binding oligomerization domain containing 1	Nod1	0.908953	1.13
Fas (TNF receptor superfamily member 6)	Fas	0.490989	1.12
Cnn2 Calponin 2	Cnn2	0.506035	1.11
Mannose-binding lectin (protein C) 2	Mbl2	0.576435	1.11
Serine (or cysteine) peptidase inhibitor, clade E, member 1	Serpine1	0.864046	1.11
Fc receptor, IgG, high affinity I	Fcgr1	0.595700	1.1
C3 Complement component 3	C3	0.932644	1.09
Transglutaminase 2, C polypeptide	Tgm2	0.942308	1.08
Fc receptor, IgG, low affinity III	Fcgr3	0.759051	1.07
Platelet/endothelial cell adhesion molecule 1	Pecam1	0.602565	1.06
Fyn proto-oncogene	Fyn	0.862847	1.05
Moesin	Msn	0.730610	1.05
Cyp2s1 Cytochrome P450, family 2, subfamily s, polypeptide 1	Cyp2s1	0.593454	1.04
V-ral simian leukemia viral oncogene homolog B (ras related)	Ralb	0.891912	1.04
Phospholipase D2	Pld2	0.888537	1.03
Dock1 Dedicator of cytokinesis 1	Dock1	0.830161	1.02
Vav 1 oncogene	Vav1	0.972614	1.02

Milk fat globule-EGF factor 8 protein	Mfge8	0.979737	1
Myeloid differentiation primary response gene 88	Myd88	0.559444	1
Calr Calreticulin	Calr	0.684084	-1.01
Csk C-src tyrosine kinase	Csk	0.735922	-1.01
Mitogen-activated protein kinase 14	Mapk14	0.714731	-1.01
RAB5A, member RAS oncogene family	Rab5a	0.994265	-1.01
RAS-related C3 botulinum substrate 2	Rac2	0.993301	-1.01
Ager Advanced glycosylation end product-specific receptor	Ager	0.906147	-1.02
CD47 antigen (Rh-related antigen, integrin-associated signal transducer)	Cd47	0.961496	-1.03
Colec12 Collectin sub-family member 12	Colec12	0.842676	-1.03
Phosphatase and tensin homolog	Pten	0.680883	-1.04
RAB7, member RAS oncogene family	Rab7	0.674196	-1.04
Adipoq Adiponectin, C1Q and collagen domain containing	Adipoq	0.675289	-1.06
Stab2 Stabilin 2	Stab2	0.635364	-1.07
Clec7a C-type lectin domain family 7, member a	Clec7a	0.822026	-1.09
Phospholipase D1	Pld1	0.549653	-1.09
Ras homolog gene family, member A	Rhoa	0.314319	-1.1
Tumor necrosis factor	Tnf	0.991142	-1.1
RAS-related C3 botulinum substrate 1	Rac1	0.398504	-1.11
Toll-like receptor 3	Tlr3	0.675539	-1.11
Mucolipin 3	Mcoln3	0.625773	-1.12
Spleen tyrosine kinase	Syk	0.639772	-1.12
Phospholipase A2, group V	Pla2g5	0.390178	-1.15
V-ral simian leukemia viral oncogene homolog A (ras related)	Rala	0.580031	-1.18
Macrophage migration inhibitory factor	Mif	0.343382	-1.24
Csf2 Colony stimulating factor 2 (granulocyte-macrophage)	Csf2	0.602286	-1.26
Interleukin 1 receptor-like 1	Il1rl1	0.188671	-1.26
Phosphatidylinositol 3-kinase, catalytic, beta polypeptide	Pik3cb	0.362926	-1.29
Phosphatidylinositol-4-phosphate 5-kinase, type 1 alpha	Pip5k1a	0.266666	-1.32
Tumor necrosis factor (ligand) superfamily, member 11	Tnfsf11	0.340306	-1.32
Protein kinase C, epsilon	Prkce	0.286300	-1.52
Scarb1 Scavenger receptor class B, member 1	Scarb1	0.448281	-1.57
CD44 antigen	Cd44	0.019882†	-1.76
Toll-like receptor 9	Tlr9	0.195746	-1.96
Sialic acid binding Ig-like lectin 1, sialoadhesin	Siglec1	0.075492	-2.53
CD36 antigen	Cd36	0.044692†	-2.95
Phospholipase A2, group IVA (cytosolic, calcium-dependent)	Pla2g4a	0.012369†	-32.39

Supplementary Table 5. Complete list of quantitative RT^{2}PCR phagocytosis arrays analysis in injured livers (48h post-TAA injection) of FVB mice compared to BALB/c. qPCR data were normalized to housekeeping gene b2MG (n=4). †, Two-tailed p value <0.05. (not adjusted, unpaired t-test).

Gene name	Gene Symbol	p-Value	Fold Regulation
Serine (or cysteine) peptidase inhibitor, clade E, member 1	Serpine1	0.003171†	27.24
Transglutaminase 2, C polypeptide	Tgm2	0.002475†	9.02
Macrophage receptor with collagenous structure	Marco	0.028877†	8.15
Cd14 CD14 antigen	Cd14	0.004156†	7.41
Integrin alpha M	Itgam	0.033442†	6.43
Toll-like receptor adaptor molecule 1	Ticam1	0.058652	3.88
Csf1 Colony stimulating factor 1 (macrophage)	Csf1	0.008903†	3.86
Platelet/endothelial cell adhesion molecule 1	Pecam1	0.015245†	3.5
Ceacam3 Carcinoembryonic antigen-related cell adhesion molecule 3	Ceacam3	0.095979	3.49
V-ral simian leukemia viral oncogene homolog B (ras related)	Ralb	0.001305†	3.04
Spleen tyrosine kinase	Syk	0.023965†	3.03
Myeloid differentiation primary response gene 88	Myd88	0.209839	2.85
Scarb1 Scavenger receptor class B, member 1	Scarb1	0.235619	2.76
Tumor necrosis factor	Tnf	0.108409	2.7
Cnn2 Calponin 2	Cnn2	0.039428†	2.69
Fyn proto-oncogene	Fyn	0.029979†	2.52
Iqsec1 IQ motif and Sec7 domain 1	Iqsec1	0.507281	2.41
Calr Calreticulin	Calr	0.046452†	2.38
Dock1 Dedicator of cytokinesis 1	Dock1	0.101461	2.37
Rap guanine nucleotide exchange factor (GEF) 3	Rapgef3	0.047993†	2.36
C3 Complement component 3	C3	0.012624†	2.35
RAS-related C3 botulinum substrate 2	Rac2	0.104589	2.24
Moesin	Msn	0.041337†	2.23
Phospholipase D2	Pld2	0.047378†	2.18
Clic4 Chloride intracellular channel 4 (mitochondrial)	Clic4	0.000860†	2.14
RAB7, member RAS oncogene family	Rab7	0.006702†	2.11
Integrin beta 2	Itgb2	0.096238	2.03
Anxa1 Annexin A1	Anxa1	0.052177	2.02
Ager Advanced glycosylation end product-specific receptor	Ager	0.045315†	2.01
Protein kinase C, epsilon	Prkce	0.205203	1.95
Stab2 Stabilin 2	Stab2	0.077354	1.92
Macrophage migration inhibitory factor	Mif	0.008623†	1.91
Phospholipase D1	Pld1	0.058285	1.89
Mucolipin 3	Mcoln3	0.003315†	1.84
Cyp2s1 Cytochrome P450, family 2, subfamily s, polypeptide 1	Cyp2s1	0.165637	1.82
Phosphatidylinositol-4-phosphate 5-kinase, type 1 alpha	Pip5k1a	0.130610	1.78
Crp C-reactive protein, pentraxin-related	Crp	0.144782	1.74
Phosphatidylinositol 3-kinase, catalytic, beta polypeptide	Pik3cb	0.116098	1.65
Interferon gamma	Ifng	0.086464	1.62
Syntaxin 18	Stx18	0.075524	1.61
Tumor necrosis factor (ligand) superfamily, member 11	Tnfsf11	0.092460	1.61
Signal-regulatory protein beta 1A	Sirpb1a	0.203378	1.59
Milk fat globule-EGF factor 8 protein	Mfge8	0.073112	1.58
Wingless-related MMTV integration site 5A	Wnt5a	0.137931	1.58
Phospholipase A2, group V	Pla2g5	0.060297	1.56
Csk C-src tyrosine kinase	Csk	0.535604	1.54
Fc receptor, IgG, low affinity III	Fcgr3	0.299197	1.53

Protein S (alpha)	Pros1	0.126104	1.5
Fc receptor, IgE, high affinity I, gamma polypeptide	Fcer1g	0.156305	1.49
Vav 1 oncogene	Vav1	0.153424	1.49
Elmo1 Engulfment and cell motility 1, ced-12 homolog (C. elegans)	Elmo1	0.181386	1.43
RAS-related C3 botulinum substrate 1	Rac1	0.026055†	1.43
Toll-like receptor 9	Tlr9	0.475994	1.43
Phosphatase and tensin homolog	Pten	0.108659	1.41
Nucleotide-binding oligomerization domain containing 1	Nod1	0.483883	1.4
Dock2 Dedicator of cyto-kinesis 2	Dock2	0.233201	1.39
Fc receptor, IgG, low affinity IIb	Fcgr2b	0.136097	1.39
Fc receptor, IgG, high affinity I	Fcgr1	0.209067	1.37
Mitogen-activated protein kinase 14	Mapk14	0.455095	1.36
C-mer proto-oncogene tyrosine kinase	Mertk	0.160571	1.36
CD47 antigen (Rh-related antigen, integrin-associated signal transducer)	Cd47	0.235011	1.3
Fas (TNF receptor superfamily member 6)	Fas	0.335456	1.29
Ras homolog gene family, member A	Rhoa	0.202890	1.26
GULP, engulfment adaptor PTB domain containing 1	Gulp1	0.531823	1.25
Integrin alpha V	Itgav	0.340763	1.21
Wiskott-Aldrich syndrome homolog (human)	Was	0.341323	1.18
Yamaguchi sarcoma viral (v-yes-1) oncogene homolog	Lyn	0.376893	1.17
RAB5A, member RAS oncogene family	Rab5a	0.872831	-1.02
Toll-like receptor 3	Tlr3	0.655386	-1.06
Axl AXL receptor tyrosine kinase	Axl	0.776765	-1.07
Colec12 Collectin sub-family member 12	Colec12	0.474530	-1.08
Surfactant associated protein D	Sftpd	0.999044	-1.08
Interleukin 1 receptor-like 1	Il1rl1	0.790309	-1.11
Crk V-crk sarcoma virus CT10 oncogene homolog (avian)	Crk	0.947102	-1.15
CD44 antigen	Cd44	0.762628	-1.17
Vesicle-associated membrane protein 7	Vamp7	0.574963	-1.2
Adipoq Adiponectin, C1Q and collagen domain containing	Adipoq	0.377958	-1.75
V-ral simian leukemia viral oncogene homolog A (ras related)	Rala	0.093623	-1.83
Clec7a C-type lectin domain family 7, member a	Clec7a	0.270562	-2.1
CD36 antigen	Cd36	0.089066	-2.41
Csf2 Colony stimulating factor 2 (granulocyte-macrophage)	Csf2	0.933070	-2.5
Mannose-binding lectin (protein C) 2	Mbl2	0.003676†	-2.58
Sialic acid binding Ig-like lectin 1, sialoadhesin	Siglec1	0.437314	-2.98
Phospholipase A2, group IVA (cytosolic, calcium-dependent)	Pla2g4a	0.009599†	-57.38

Supplementary Table 6. Validation of differentially regulated genes from quantitative SYBR green RT^{2}PCR phagocytosis array using TaqMan RT-PCR method. All data are relative abundance of transcripts relative to housekeeping gene b2MG, expressed as mean and SEM (n=4).†, p value <0.05; ††, p value <0.01; †††, p value <0.001 compared to FVB strain at the same condition (healthy or 48h post-TAA injury, t-test).*, p value <0.05; **, p value <0.01; ***, p value <0.001 compared to healthy control of respective strain (two-tailed, not adjusted, unpaired t-test).

**Gene symbol**	**FVB (healthy)**	**BALB/c (healthy)**	**FVB (post-injury)**	**BALB/c (post-injury)**
**Serpine1**	0.0237±0.004479	0.0241±0.008699	2.033±0.2986***	0.1866±0.05270*†††
**Tgm2**	0.2023±0.03317	0.2680±0.0588	1.528±0.2270**	0.4678±0.05360*††
**Cd14**	0.1983±0.07216	0.0894±0.007788	3.040±0.5709**	0.4465±0.09379**††
**Csf1**	0.4238±0.05067	0.4085±0.1239	0.8755±0.1612*	0.3678±0.009801†
**MARCO**	0.3053±0.1101	0.05516±0.005412	2.441±0.7909*	0.2355±0.08370†
**CD36**	0.2533±0.07204	0.5610±0.1996	1.137±0.7474	1.007±0.1672
**C3**	0.3336±0.1594	1.100±0.8419	0.6473±0.1463	0.2653±0.06738
**Siglec1**	0.2810±0.04074	0.8242±0.3607	0.8175±0.5910	0.7138±0.05444
**Pla2g4a**	0.4945±0.1532	0.3345±0.04989	1.417±1.000	0.7728±0.1322*
**Mbl2**	0.6930±0.1333	0.5745±0.03267	0.5133±0.2285	1.207±0.3066
