# Supplementary material for: Hepatocyte mitochondria-derived danger signals directly activate hepatic stellate cells and drive progression of liver fibrosis
Source: Nat Commun. 2020 May 12;11:2362. doi: 10.1038/s41467-020-16092-0 (PMC7217909; doi:10.1038/s41467-020-16092-0)
Supplement: Supplementary file 3 — Reporting Summary [file 41467_2020_16092_MOESM3_ESM.pdf]

nature research

Corresponding author(s): Yury Popov

Last updated by author(s): Mar 20, 2020

Reporting Summary

Nature Research wishes to improve the reproducibility of the work that we publish. This form provides structure for consistency and transparency in reporting. For further information on Nature Research policies, see Authors & Referees and the Editorial Policy Checklist .

Statistics

For all statistical analyses, confirm that the following items are present in the figure legend, table legend, main text, or Methods section.

n/a	Confirmed	
☐	☒	The exact sample size (n) for each experimental group/condition, given as a discrete number and unit of measurement
☐	☒	A statement on whether measurements were taken from distinct samples or whether the same sample was measured repeatedly
☐	☒	The statistical test(s) used AND whether they are one- or two-sided<br>*Only common tests should be described solely by name; describe more complex techniques in the Methods section.*
☒	☐	A description of all covariates tested
☐	☒	A description of any assumptions or corrections, such as tests of normality and adjustment for multiple comparisons
☐	☒	A full description of the statistical parameters including central tendency (e.g. means) or other basic estimates (e.g. regression coefficient) AND variation (e.g. standard deviation) or associated estimates of uncertainty (e.g. confidence intervals)
☐	☒	For null hypothesis testing, the test statistic (e.g. F, t, r) with confidence intervals, effect sizes, degrees of freedom and P value noted<br>*Give P values as exact values whenever suitable.*
☒	☐	For Bayesian analysis, information on the choice of priors and Markov chain Monte Carlo settings
☒	☐	For hierarchical and complex designs, identification of the appropriate level for tests and full reporting of outcomes
☒	☐	Estimates of effect sizes (e.g. Cohen's d, Pearson's r), indicating how they were calculated

Our web collection on statistics for biologists contains articles on many of the points above.

Software and code

Policy information about availability of computer code

Data collection	no software was used for data collection
Data analysis	Data were analyzed using Excel Office 365 and GraphPad Prizm 8.4.0. Analysis of phagocytosis array data was analyzed using QIAGEN's GeneGlobe Data Analysis Center online tool according to assay's manufacturer's protocol. Primers were designed using Primer Express Software version 3.0. No custom code was used in the manuscript.

For manuscripts utilizing custom algorithms or software that are central to the research but not yet described in published literature, software must be made available to editors/reviewers. We strongly encourage code deposition in a community repository (e.g. GitHub). See the Nature Research guidelines for submitting code & software for further information.

Data

Policy information about availability of data

All manuscripts must include a data availability statement. This statement should provide the following information, where applicable:

- Accession codes, unique identifiers, or web links for publicly available datasets
- A list of figures that have associated raw data
- A description of any restrictions on data availability

The source data underlying Figures 1B, 1C, 2C, 3B-D, 4C-D, 5A, 5D-G, 6B-C, 6F-H, 7A-D, Supplementary Figures 1B, 2B, 3A, 5B, 6B, 7B-E, 8A-B are provided as a Source Data file.

Field-specific reporting

Please select the one below that is the best fit for your research. If you are not sure, read the appropriate sections before making your selection.

☒ Life sciences ☐ Behavioural & social sciences ☐ Ecological, evolutionary & environmental sciences

For a reference copy of the document with all sections, see nature.com/documents/nr-reporting-summary-flat.pdf

Life sciences study design

All studies must disclose on these points even when the disclosure is negative.

Sample size	Animal experiments were sized according to prior experience of us and others to assess the effect on liver fibrosis in mouse hepatotoxin-induced models (Popov et al. Gastroenterology. 2011 May;140(5):1642-52; Liu et al. FASEB J. 2016 Apr;30(4):1599-609). Human study was retrospective (utilizing tissue biobank). In pilot cohort I, samples (n=27) were selected to enrich the number of patients with advanced fibrosis (F3/4 stage, which are usually rare). In validation cohort II (n=114), all sequential samples available to us were analysed (n=114).
Data exclusions	No data were excluded from analysis, with the exception of rare few where technical accuracy of measurement was in question (e.g. negative value) and only if the measurement could not be repeated due to sample condition or availability. No exculsion criteria were pre-established.
Replication	All in vitro experiments were reproduced at least once using a different primary isolate of cells, as described in the manuscript. Animal experiments were performed on two or more occasions with similar results and data combined for analysis.
Randomization	Allocation of mice to experiments was random throughout the study. In human sample analysis, all available consecutive patient samples from the registry were included, retrospectively and without any selection or control for co-variates.
Blinding	Lead author was not blinded to animal experiment groups, but the other scientists who performed sample analysis were blinded and only aware of animal coded ID and not a treatment. For human study part, all scientists who performed the measurements and analysis were blinded except ML (who provided coded samples but did not take part in analysis) and received clinical information for analysis only after all data were collected and "locked".

Reporting for specific materials, systems and methods

We require information from authors about some types of materials, experimental systems and methods used in many studies. Here, indicate whether each material, system or method listed is relevant to your study. If you are not sure if a list item applies to your research, read the appropriate section before selecting a response.

Materials & experimental systems

n/a	Involved in the study	
☐	☒	Antibodies
☒	☐	Eukaryotic cell lines
☒	☐	Palaeontology
☐	☒	Animals and other organisms
☐	☒	Human research participants
☐	☒	Clinical data

Methods

n/a	Involved in the study	
☒	☐	ChIP-seq
☒	☐	Flow cytometry
☒	☐	MRI-based neuroimaging

Antibodies

Antibodies used	All antibody used are specified in Table S2
Validation	In this study, we only used well characterized antibody that are extensively validated and commonly used in the field. Additionally, specificity of immunostainings performed in our lab are routinely validated by omission of primary antibody and in tissues of knockout animals, if available.

Animals and other organisms

Policy information about studies involving animals; ARRIVE guidelines recommended for reporting animal research

Laboratory animals	Male laboratory inbred mice of 6-7 weeks of age were used (strains FVB/NJ, C57BL/6J, BALB/c).
Wild animals	no wild animals were used in the study
Field-collected samples	no field-collected samples were used in the study

Ethics oversight	Animal experiments were reviewed and approved by Beth Israel Deaconess Medical Center's Institutional Animal Care and Use Committee (IACUC).

Note that full information on the approval of the study protocol must also be provided in the manuscript.

Human research participants

Policy information about studies involving human research participants

Population characteristics	Adult (age 18 and over) patients with NAFLD getting a liver biopsy. Mean age was 52.4±12.9 (range 21-69) in cohort I and 56.4 ±12.3 (range 30-90) in cohort II. Sex ratio (male/female) was 14/13 in cohort I and 72/42 in cohort II.
Recruitment	All patients with suspected NAFLD getting a liver biopsy were recruited from 2009 to 2013. Only patients who agreed to liver biopsy were included because studied parameter was assessed against histological parameters on biopsy, which may have introduced a bias toward selection of more advanced liver disease. Because our study focused on non-alcoholic steatohepatitis (NASH), an advanced form of non-alcoholic fatty liver disease (NAFLD), this selection represents a real world population in most need of diagnostics and treatment, and is unlikely to diminish clinical significance or the findings in relation to general NAFLD patients pool.
Ethics oversight	The study was reviewed and approved by the Institutional Review Board of Beth Israel Deaconess Medical Center and informed consent was obtained from all study participants.

Note that full information on the approval of the study protocol must also be provided in the manuscript.

Clinical data

Policy information about clinical studies

All manuscripts should comply with the ICMJEguidelines for publication of clinical research and a completedCONSORT checklist must be included with all submissions.

Clinical trial registration	This was not a clinical trial but an analysis of specimen from biobank. We provide Tier 1 and 2 sample information according to BRISQ reporting guidelines (Biospecimen Reporting for Improved Study Quality)
Study protocol	no study protocol was established for this retrospective analysis of consecutive patient samples.
Data collection	Patients were recruited and enrolled between 2009 and 2013. Data was collected from medical records and from patient questionnaires. Follow-up data collection continued until presently.
Outcomes	this was a registry study so there was no predefined outcomes
